# A Medical Causerie

**Published:** 1916-03-25

**Authors:** 


### A MEDICAL CAUSERIE.

In the Metropolitan district there was, some little time ago, a cry of despair on the announcement that sick and wounded soldiers were no longer to be sent to the civil hospitals. Impetuous critics flew at the War Office and denounced the authorities in vigorous terms. Apparently some writers had not recognised that every bed given to the Army was a bed subtracted from the civil population. Such a course may have been in the early days of the war a necessity of the position; but if so, it was a matter for regret rather than for rejoicing. At the outset every civil hospital—or at least the majority of them—seemed to think that advertisement and prestige were to be obtained from the reception of the wounded, and in more than one instance the War Office was actually entreated to supply at least a few. One institution was credited with a sharp manœuvre by which it managed to deflect to itself a convoy intended for a rival institution. Whether military hospitals existed, or ought to have existed, and whether justice was being secured for the civil population, were questions which hardly attracted attention. In short, the War Office found the civil hospitals not only willing, but anxious to relieve them of part of their official responsibilities. At first this state of matters was perhaps pardonable; but it ought long since to have been made quite unnecessary. To secure such an end, however, pressure and resistance from the hospital authorities were necessary conditions, and these, it is to be feared, have been sadly lacking. In one of the Northern cities there were at one time literally hundreds of empty beds in the military hospitals while soldiers were being sent into the three general hospitals founded and supported for the benefit of the sick poor. Not only so, but the fact of such admissions was quoted on behalf of an appeal for the support of a Red Cross hospital, which enjoyed the patronage of the local leaders of society. And while all this was going on there were wards in a neighbouring military hospital actually closed for lack of patients. Of course this sort of thing is little short of scandalous, but it is likely to continue and to be repeated so long as the civil hospitals hold a complaisant tone to the War Office, and the care of the wounded is raised to the dignity of a social function. Even now, after more than eighteen months of war, the authorities are once more invading the civil hospitals in the Metropolitan district. Everybody agrees that the soldiers ought to have—as they deserve to have—the best possible medical and surgical care. But by organisation and foresight this would have been readily possible without sacrifice of the interests of the civil population.

---

The term "accident" has in recent years received under the influence of legal decisions a very extensive application, and quite recently it has been ruled that "rheumatism" produced by a wetting incurred by a workman is in the legal sense an "accident." In these circumstances it seems rather hard that a plaintiff who, in walking along the street, slipped on a piece of orange-peel, and as a result fell and broke his thigh, should find his claim refused. The man had gone ashore on the business of the ship of which he was the skipper, and in order to return had to pass along a street where there were a number of fruit stalls; and here the catastrophe occurred. The County Court judge decided for the plaintiff on the ground that the accident arose "out of his employment," seeing that in the particular street by which he had to pass he was more exposed to the dangers of orange-peel than were ordinary persons in ordinary parts of the Metropolis. But this conclusion was rejected by the Court of Appeal, where it was held that the accident, though occurring in the course of the man's employment, did not "arise out of it," seeing that the orange-peel peril was common to all the citizens. There are now so many medical men engaged upon insurance work that the decision of the Courts in questions of this kind are of special interest to the medical profession.

---

The critics of the medical officers engaged in the examination of recruits have surely damaged their position by instructing a member of Parliament to ask in the House of Commons a question which suggested that men who had artificial limbs had been passed as fit for military service, and that the same fate had been awarded to others so deficient in mental capacity that they were unable to understand or to answer the questions addressed to them. The discovery of mares' nests is a fruitful occupation of the day and hour, but there are limits even to the credulity of the public. Mr. Tennant quite properly refused to take such suggestions seriously, and, speaking of the medical officers, he added, "I think that, instead of being held up to obloquy, they deserve support and recognition in the heavy work they are patriotically doing on behalf of the country." This is a generous and well-deserved tribute, and in face of it no further notice need be taken of critics who must have sensation whatever the cost. The fact is that numbers of medical men who have undertaken recruiting work have done so to the prejudice of their own private interests and purely from patriotic motives. In at least one district all the fees received for this work were sent as a donation to the Belgian Doctors' and Pharmacists' Fund. Of course mistakes have been made, and possibly some of these have been rather startling. But often the conditions under which the work had to be done were most unsatisfactory from a professional point of view, and, further, it must be remembered that there are cases in which there is room for a legitimate difference of opinion. Even highly trained physicians, for example, do not always agree as to the exact significance to be attached to so definite an event as a cardiac murmur.

